# Sex-Related Differences in the Association between Metabolic Syndrome and Gallstone Disease

**DOI:** 10.3390/ijerph18041958

**Published:** 2021-02-18

**Authors:** Francesco Baratta, Daniele Pastori, Nicholas Cocomello, Alessandra Colantoni, Domenico Ferro, Francesco Angelico, Maria Del Ben

**Affiliations:** 1 Department of Clinical, Internal, Anaesthesiological and Cardiovascular Sciences, Sapienza University of Rome, 00161 Rome, Italy; francesco.baratta@uniroma1.it (F.B.); daniele.pastori@uniroma1.it (D.P.); nicholas.cocomello@gmail.com (N.C.); alessandracolantoni@libero.it (A.C.); domenico.ferro@uniroma1.it (D.F.); 2 Department of Public Health and Infectious Diseases, Sapienza University of Rome, 00161 Rome, Italy; francesco.angelico@uniroma1.it

**Keywords:** metabolic syndrome, gallstone disease, cholecystectomy, risk factor, epidemiology, cholecystectomy

## Abstract

Metabolic syndrome (MetS) and gallstone disease (GD) share common risk factors. Several epidemiological studies reported that subjects with Mets are more likely to have GD than those without and that cholecystectomy (CHO) may increase the risk of MetS. The aim of the study was to evaluate the association between MetS and GD in a large cohort of patients with metabolic risk factors in Italy. The study was performed in 620 consecutive outpatients referring to the University outpatients’ clinic for the management of cardiovascular risk factors. MetS were diagnosed according to the ATPIII Expert Panel modified criteria. GD was defined as gallstones documented by abdominal ultrasound (US) or previous cholecystectomy. The prevalence of GD was significantly higher in women than in men (22.3% vs. 13.1%, *p* = 0.003). Both prevalence of GD (17.1% vs. 8.4%, *p* = 0.015) and CHO (9.0% vs. 1.7%, *p* = 0.002) were significantly higher in males with MetS as compared to those without. By contrast, the prevalence of GD and of CHO was similar in women with and without MetS. After correction for confounders, MetS was an independent predictor of both GD (odds ratio (OR) 1.943, *p* = 0.048) and CHO (OR 5.075, *p* = 0.011) in men, but not in women. In conclusion, in this study, including western subjects with cardiometabolic risk factors, the association between GD, prior CHO and MetS were found in men, but not in women.

## 1. Introduction

Metabolic syndrome (MetS) is a common clinical condition affecting approximately 20% of the non-diabetic population, rising up to 40% of the population over 50 years of age in western countries [1].

MetS occurs in association with a cluster of major cardiovascular risk factors and metabolic abnormalities, including impaired fasting glucose, glucose intolerance or diabetes, insulin resistance, pro-atherogenic dyslipidemia (i.e., increased levels of triglycerides and decreased HDL-cholesterol), arterial hypertension and central and overall obesity [2,3]. MetS is strongly associated with an increased risk of cardiovascular disease and type 2 diabetes [4]. Thus, the annual rate of cardiovascular events in patients with MetS is 0.9–2.1%, with a two-fold risk in comparison with those without MetS [5,6].

Gallstone disease (GD) is a common disease in most developed countries and a frequent cause of abdominal surgery [7,8,9,10,11]. A trend for increasing gallstone prevalence has been identified in Europe and North America [12]. Most people with gallstones (about 80%) never have symptoms throughout life [13]. The “five f’s” definition well describes the usual patient with GD: “fair, fat, forty, fertile and female”, i.e., an overweight middle-aged white woman with a history of several pregnancies [14]. However, GD is a multifactorial disease and risk factors are still poorly understood.

MetS and GD share common risk factors, the most relevant being represented by abdominal obesity and insulin resistance, which both have been associated with increased body cholesterol synthesis, hypersecretion of biliary cholesterol and higher bile lithogenicity [15,16]. Therefore, the hypothesis that GD is another component of MetS has been suggested [17].

Several cross-sectional and case-report studies reported that subjects with GD are more likely to have MetS than those without GD and that cholecystectomy (CHO) may increase the risk of MetS [17,18,19,20,21]. On the other hand, an increased prevalence of GD has been reported in subjects with MetS, where the prevalence of GD in women who had five components of MetS was five times higher than in those without any MetS component [22].

More than 85% of gallstones in Western countries are cholesterol or mixed stones with cholesterol as the main component, for which biliary cholesterol supersaturation may play a role for gallstone formation [13,23]. However, most data on the association of MetS with GD refer to studies performed in the Far East countries, where the occurrence of pigmented stones is more common, and risk factors may be different from Western countries [24].

The aim of the study was to evaluate the association between MetS and GD in a large cohort of patients with metabolic risk factors in Italy who underwent abdominal ultrasound (US) examination for the examination of the presence of fatty liver in the PLINIO study (NCT04036357) [25,26]. In addition, we investigated the presence of sex-related differences in the association between gallstone disease and MetS.

## 2. Methods

The study was performed on 620 consecutive outpatients referring to the Day Service of Internal Medicine and Metabolic Diseases of the Policlinic Umberto I University Hospital in Rome. All patients had ≥1 of the following cardiovascular risk factors to be included in the study: type 2 diabetes, arterial hypertension, overweight/obesity, dyslipidemia, MetS.

Waist circumference, height and weight were recorded with subjects wearing light clothing, without shoes and body mass index (BMI) was calculated as weight (kg) divided by height (m^2^). Arterial blood pressure was measured on the right arm with the subjects in a sitting position and after a 5 min rest, using a mercury sphygmomanometer: the average of two measurements, 1 min apart, was considered. Metabolic syndrome was diagnosed according to the modified criteria of the ATP III Expert Panel of the US-NCEP (United States National Centers for Envi-ronmental Prediction) [27]. Diabetes was diagnosed according to the WHO (World Health Organization) criteria. Subjects taking insulin or oral antidiabetic drugs were considered to have diabetes. Subjects underwent a routine biochemical evaluation, including serum liver enzymes, fasting total and HDL-cholesterol, triglycerides, glucose and insulin. An Olympus AN-560 apparatus (Olympus Optical Co., Ltd., Tokyo, Japan) using an enzymatic colorimetric method measured total serum cholesterol, HDL-cholesterol and triglycerides. LDL-cholesterol levels were calculated according to the Friedewald formula. Plasma insulin levels were assayed commercially by available radioimmunoassay. The homeostasis model of insulin resistance (HOMA-IR) was calculated and used as a measure of IR (insulin resistance). Insulin resistance was arbitrarily defined as HOMA-IR values in the top quartile (>5).

Written consent was obtained from all subjects before the study, and the study conforms to the ethical guidelines of the 1975 Declaration of Helsinki. The Ethics committee of the Policlinic Umberto I Hospital of Rome (ref. n° 2277/2011) approved the study. The manuscript was prepared and revised according to the STROBE Statement.

### 2.1. Ultrasonography Examination (US)

Liver US scanning was performed in all patients who have been fasting for at least 8 h, using a GE Vivid S6 apparatus (General Electric Company, Boston, MA, USA) equipped with a convex 3.5 MHz probe. GD was defined as gallstones documented by the US or evidence of a previous CHO. Patients with incident gallbladder diseases other than gallstones were excluded from the study.

### 2.2. Statistical Analysis

Continuous variables are reported as mean ± standard deviation or median with interquartile range. Student’s *t*-test or Mann–Whitney test, depending on their distribution, analyzed continuous variables. Group comparisons were performed by ANOVA or Kruskal–Wallis, when appropriate. Dichotomous variables are reported as numbers and percentages. Differences were tested using the χ2 test for categorical variables. Pearson’s *r* coefficients were calculated for bivariate correlations. All tests were two-tailed, and a *p* < 0.05 was considered as the cutoff for statistical significance. We performed a descriptive analysis of the characteristics of patients affected by GD and/or CHO. We performed a descriptive analysis of the characteristics of patients affected by GD and/or CHO. Univariate and multivariate logistic regression analyses were performed to estimate the association of GD and CHO with MetS. To perform the multivariate model, variables presenting significant coefficients at univariate analysis were used as covariates after testing for collinearity. Finally, the same multivariate analysis was performed in men and women separately to underline sex differences in the association between GD, CHO and MetS. Statistical analysis was performed by using the SPSS statistical software version 20.0 for Windows (SPSS, Inc., Chicago, IL, USA).

## 3. Results

The mean age was 54.9 ± 11.8 years; 37.6% were women, and 54.5% had metabolic syndrome.

Prevalence of GD (gallstones plus CHO) was significantly higher (*p* = 0.003) in women (22.2%) than in men (13.1%). Prevalence of previous CHO was three times as high (*p* < 0.001) in females (15.5%) as compared to males (5.7%).

The prevalence of MetS was similar in both sexes (54.2% and 55.4% in males and females, respectively). Clinical and biochemical characteristics of patients with and without MetS according to sex are reported in Table 1. Subjects with MetS had a significantly higher prevalence of all components of MetS. Moreover, they also had significantly increased mean HOMA-IR and age.

The prevalence of GD was significantly higher in males with MetS as compared to those without (17.1% versus 8.4%; *p* = 0.016). Similarly, prevalence of previous CHO was significantly increased in males with MetS (9.0% vs. 1.7%; *p* = 0.002). By contrast, the prevalence of GD and of CHO was similar in women with and without MetS.

**Figure 1:**
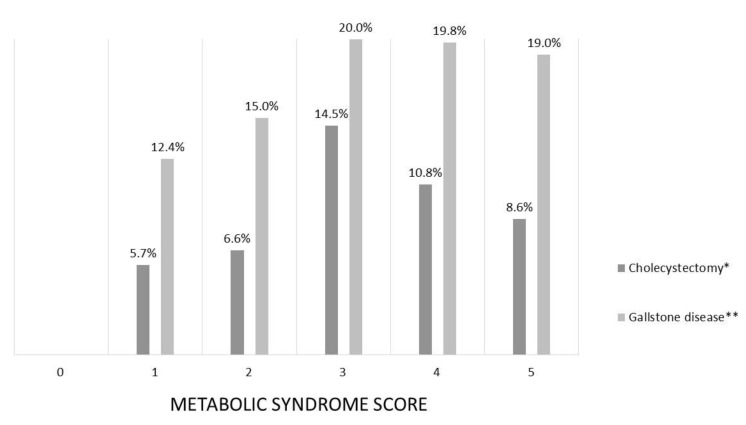
Prevalence of cholecystectomy and gallstone disease according to metabolic syndrome score. (* *p* = 0.044, ** *p* = 0.014).

In the whole cohort, the prevalence of previous CHO and GD significantly increased with the number of the components of MetS (*p* = 0.044 and *p* = 0.014, respectively) (Figure 1). The prevalence of GD was 19.0% in subjects who had five components of MetS, while no cases of GD were observed in subjects without any of the components of MetS.

Prevalence of MetS was significantly higher in subjects who underwent CHO (72.4% vs. 52.9%; *p* = 0.005) and in those with GD (65.0% vs. 52.4%; *p* = 0.023). Mean HOMA-IR was similar in subjects with GD and in those without gallstones and evidence of a previous CHO (4.22 ± 3.19 and 4.65 ± 4.02, *p* = 0.336, respectively). Prevalence of CHO and of GD did not differ in subjects in the top quartile of HOMA-IR (insulin resistance).

As reported in Table 2 and Table 3, at univariate analysis, age over 65 years, female sex, central obesity, high blood pressure and MetS were significantly associated with GD, while at multivariate analysis, only age and female sex were independently associated. By contrast, at univariate analysis, CHO was strongly correlated with increasing age, female sex and the presence of MetS, while at multiple logistic analysis age, female **sex** and MetS were independent predictors after controlling for confounding factors. Separate univariate analyses conducted in men and women are reported in Supplementary Tables S1 and S2. In a further multiple logistic analysis of factors associated with CHO, where its clinical components were introduced instead of Mets, none of these were independently correlated (data not shown).

Finally, in separate multivariate analyses by sex, MetS was the only independent predictor of GD and CHO in males (OR = 1.943, *p* = 0.048 and OR = 5.075, *p* = 0.011, respectively) while age above median was in females (OR = 2.230, *p* = 0.011 and OR = 3.044, *p* = 0.005, respectively) (Table 4).

## 4. Discussion

We found a significant association between MetS and GD in male patients affected by cardiometabolic diseases.

Few studies, so far, have addressed the association between MetS and GD, and most have been performed in Far East countries, where pigmented stones are more common, and the prevalence of GD is similar in men and women [20,21,22,23]. In our study, according to most epidemiological data in Western countries [8,9,11,12], the prevalence of GD was higher in women as compared to men. In agreement with previous studies [19,20], we found a significant association between MetS and GD. Men with MetS had GD two times more often than patients without Mets, while similar proportions were observed in women with and without MetS. This is in agreement with a longitudinal study performed in China where a significant association was observed between MetS and GD events for males but not for females [28]. Sex differences have also been reported in other studies. In a cross-sectional study in Taiwan where MetS and GD were associated, lower HDL-C was the most important metabolic factor for GSD in men, whereas, in women, abdominal obesity had a higher odds ratio for GD [20].

As for the pathophysiological mechanisms underlying the positive association between MetS and GD, we support the hypothesis that visceral obesity and hepatic insulin resistance may play a central role in promoting cholesterol bile supersaturation and gallstone formation. This hypothesis is supported by a study showing an association of GD with insulin resistance and MetS in a high-risk Hispanic population [17] and by a cross-sectional study carried out in Korea where insulin resistance was associated with GD in postmenopausal women with abdominal obesity [16]. Moreover, it has been observed that mice with isolated hepatic insulin resistance created by liver-specific disruption of the insulin receptor are markedly predisposed towards cholesterol gallstone formation [15].

In our study, MetS were common among subjects with GD and particularly frequent among those with previous CHO, where three-quarters had MetS. At multivariate analysis, CHO was strongly associated with MetS. These findings are in keeping with a study performed in Turkey, where MetS were associated with complicated GD and suggested as a further indication for prophylactic surgery in patients with GD [19]. Moreover, in a Chinese population, the prevalence of MetS was significantly higher in subjects with a history of CHO (63.5%) than in those with gallstones (47.0%) or in those without gallstone disease [21]. Several risk factors are probably involved in the increased risk for MetS after biliary surgery, but so far, the underlying mechanisms are not fully elucidated. Recently, the tendency for an increase in body weight after CHO, because of the return to eating habits that preceded the intervention, was suggested as a possible contributing factor for the increased incidence of MetS after biliary surgery [18]. In particular, an increased incidence of metabolic abnormalities among cholecystectomized patients, such as hyperlipidemia and hyperglycemia, was reported [29]. However, in our study, none of the individual features of MetS was independently associated with CHO when MetS were replaced by its clinical components.

This study has some limitations. First, the study cohort was recruited from individuals attending the university clinic for the presence of at least one major cardiovascular risk factor and, therefore, may not represent the general population. A further limitation is that this is a cross-sectional study, and no causal relationships can be assessed.

However, the study also has some strengths. All the enrolled patients underwent the abdominal US, and the presence of gallstone and the evidence of previous CHO were documented by imaging and not self-reported by patients or extracted by clinical code databases. In addition, this is a study conducted on a large western cohort, while all recent data on gallstone disease and MetS come mainly from Asian patients.

## 5. Conclusions

In summary, in this large western population of subjects with cardiometabolic risk factors, the prevalence of GD and of previous CHO was significantly increased in males with MetS. This finding is consistent with the hypothesis of cholesterol bile supersaturation and increased risk for gallstone formation in subjects with MetS. Moreover, we also found a high prevalence of MetS among subjects with previous CHO. Therefore, the relationship between MetS and GD seems to be bidirectional, with numerous predisposing factors in both directions. The increased risk for MetS after CHO is still under debate and should be confirmed by a prospective study to evaluate the predictive value of biliary surgery on the development of MetS. Further studies are needed to better understand the different associations between MetS and GD according to sex.

## Figures and Tables

**Table 1:** Clinical and biochemical variables in males and females with and without metabolic syndrome (MetS).

Variables	Males			Females		
	MetS N = 210	No MetS N = 170	*p*-Value	MetS N = 129	No MetS N = 105	*p*-Value
Age (y)	57.7 ± 11.2	53.2 ± 13.7	<0.001	59.5 ± 11.3	56.2 ± 12.8	0.020
BMI (kg/m^2^)	31.2 ± 4.3	27.7 ± 4.1	<0.001	31.2 ± 5.8	27.5 ± 4.7	<0.001
Waist circumference (cm)	111.9 ± 10.5	101.5 ± 11.2	<0.001	105.3 ± 11.5	96.8 ± 11.3	<0.001
Arterial hypertension (%)	49.8	72.5	<0.001	40.9	72.6	<0.001
Diabetes (%)	38.6	8.9	<0.001	45.9	9.5	<0.001
Fasting blood glucose (mg/dL)	114.0 ± 32.9	94.9 ± 15.7	<0.001	111.8 ± 31.0	90.3 ± 19.1	<0.001
HOMA-IR	5.5 ± 4.1	2.7 ± 1.5	<0.001	4.7 ± 2.7	2.7 ± 2.0	<0.001
HDL-cholesterol (mg/dL)	40.9 ± 10.8	49.8 ± 10.2	<0.001	50.6 ± 12.6	64.2 ± 15.6	<0.001
Triglycerides (mg/dL)	201.5 ± 140.7	125.0 ± 66.6	<0.001	152.9 ± 70.8	102.2 ± 41.4	<0.001
Uric acid (mg/dL)	6.4 ± 1.3	5.8 ± 1.2	<0.001	6.4 ± 1.3	5.9 ± 1.2	<0.001
AST (U/L)	26.2 ± 14.9	24.0 ± 14.4	0.109	26.2 ± 15.0	24.0 ± 14.4	0.780
ALT (U/L)	37.1 ± 27.0	31.4 ± 25.0	0.005	37.1 ± 27.0	31.4 ± 25.0	0.238
GGT (U/L)	46.8 ± 48.5	41.9 ± 72.5	0.403	46.8 ± 48.5	41.9 ± 72.6	0.877
Gallstones (%)	8.1	6.7	0.700	6.2	7.6	0.796
Cholecystectomy (%)	9.0	1.7	0.002	17.8	12.5	0.280
Gallstone disease (%)	17.1	8.4	0.015	24.0	20.0	0.528

BMI = Body mass index; HOMAR-IR = Homeostasis model of insulin resistance; HDL-cholesterol = High-density lipoprotein-cholesterol; AST = Aspartate aminotransferase; ALT = Alanine aminotransferasel; GGT = Gamma-glutaryl aminotransferase.

**Table 2:** Univariate analysis of clinical and laboratory variables associated with gallstone disease and cholecystectomy.

Variables	Gallstone Disease		Cholecystectomy	
	OR (95% CI)	*p*-Value	OR (95% CI)	*p*-Value
**Age (y)**	1.038 (1.018–1.058)	<0.001	1.045 (1.020–1.072)	<0.001
**Female gender**	1.917 (1.261–2.914)	0.002	3.131 (1.815–5.402)	0.002
**Obesity (BMI ≥ 30 kg/m^2^)**	1.522 (1.018–2.368)	0.041	1.649 (0.962–2.824)	0.069
Central obesity (high waist circumference) *	1.543 (0.939–2.535)	0.087	1.728 (0.915–3.264)	0.092
Impaired fasting glucose *	1.483 (0.972–2.265)	0.068	1.495 (0.871–2.565)	0.145
Low HDL *	1.057 (0.679–1.644)	0.807	1.460 (0.844–2.526)	0.176
High triglycerides *	0.983 (0.637–1.519)	0.983	0.789 (0.448–1.391)	0.414
**Total cholesterol (mg/dL)**	1.000 (0.995–1.005)	0.999	0.999 (0.993–1.006)	0.782
**LDL cholesterol (mg/dL)**	1.000 (0.994–1.005)	0.871	1.000 (0.992–1.007)	0.902
High blood pressure *	1.908 (1.133–3.213)	0.015	1.861 (0.943–3.670)	0.073
**Metabolic syndrome**	1.690 (1.088–2.625)	0.019	2.335 (1.283–4.251)	0.006
**High HOMA-IR**	0.966 (0.615–1.158)	0.880	1.153 (0.655–2.030)	0.622
High ALT **	0.858 (0.547–1.346)	0.504	1.127 (0.648–1.962)	0.671
High AST **	1.050 (0.584–1.886)	0.871	1.681 (0.870–3.247)	0.122
High GGT **	1.229 (0.785–1.924)	0.367	1.693 (0.979–2.929)	0.059

* = defined according to ref. [26]; ** = values above median; CI = confidence interval; OR = odds ratio.

**Table 3:** Factors associated with gallstone disease and cholecystectomy al multiple logistic regression analysis.

Variables	Gallstone Disease		Cholecystectomy	
	OR (95% CI)	*p*-Value	OR (95% CI)	*p*-Value
Age above median	2.074 (1.313–3.276)	0.002	2.573 (1.376–4.811)	0.003
Female sex	1.795 (1.164–2.796)	0.008	2.891 (1.639–5.101)	<0.001
Metabolic syndrome	1.446 (0.918–2.278)	0.112	2.001 (1.079–3.713)	0.028

**Table 4:** Multiple logistic regression analysis of factors associated with gallstone disease and cholecystectomy in males and females.

**Gallstone Disease**				
**Variables**	Male		Female	
	**OR (95% CI)**	***p*-Value**	**OR (95% CI)**	***p*-Value**
Age above median	1.801 (0.969–3.349)	0.063	2.230 (1.173–4.241)	0.014
Metabolic syndrome	1.943 (1.007–3.748)	0.048	1.079 (0.566–2.057)	0.818
**Cholecystectomy**				
**Variables**	Male		Female	
	**O (95% CI)**	***p*-Value**	**OR (95% CI)**	***p*-Value**
Age above median	1.619 (0.648–4.045)	0.302	3.044 (1.403–6.604)	0.005
Metabolic syndrome	5.075 (1.448–17.793)	0.011	1.240 (0.580–2.649)	0.579

## Data Availability

The data presented in this study are available on request from the corresponding author. The data are not publicly available due to privacy.

## Supplementary Materials

The following are available online at https://www.mdpi.com/1660-4601/18/4/1958/s1, Table S1: Univariate analysis of clinical and laboratory variables associated with gallstone disease and cholecystectomy in men, Table S2: Univariate analysis of clinical and laboratory variables associated with gallstone disease and cholecystectomy in women.

## Abbreviations

US	Abdominal ultrasound
ATP III	Adult treatment panel
ALT	Alanine aminotransferase
AST	Aspartate aminotransferase
BMI	Body mass index
CHO	Cholecystectomy
MetS	Metabolic syndrome
GD	Gallstone disease
GGT	Gamma-glutaryl aminotransferase
HDL	High-density lipoprotein
HOMA-IR	Homeostasis model of insulin resistance
LDL	Low-density lipoprotein
US-NCEP	United States National Centers for Environmental Prediction
WHO	World Health Organization

## References

[1] Cameron A.J., Shaw J.E., Zimmet P.Z. (2004). The metabolic syndrome: Prevalence in worldwide populations. Endocrinol. Metab. Clin. N. Am..

[2] Eckel R.H., Grundy S.M., Zimmet P.Z. (2005). The metabolic syndrome. Lancet.

[3] National Cholesterol Education Program (NCEP) Expert Panel on Detection, Evaluatuin, and Treatment of High Blood Cholesterol in Adults (Adult Treatment Panel III) (2002). Third report of the national cholesterol education program (ncep) expert panel on detection, evaluation, and treatment of high blood cholesterol in adults (adult treatment panel iii) final report. Circulation.

[4] Mottillo S., Filion K.B., Genest J., Joseph L., Pilote L., Poirier P., Rinfret S., Schiffrin E.L., Eisenberg M.J. (2010). The metabolic syndrome and cardiovascular risk. J. Am. Coll. Cardiol..

[5] Khosravi-Boroujeni H., Ahmed F., Sadeghi M., Roohafza H., Talaei M., Dianatkhah M., Pourmogaddas A., Sarrafzadegan N. (2015). Does the impact of metabolic syndrome on cardiovascular events vary by using different definitions?. BMC Public Health.

[6] Santaniemi M., Ukkola O., Malo E., Bloigu R., Kesäniemi Y.A. (2014). Metabolic syndrome in the prediction of cardiovascular events: The potential additive role of hsCRP and adiponectin. Eur. J. Prev. Cardiol..

[7] Aerts R., Penninckx F. (2003). The burden of gallstone disease in Europe. Aliment. Pharmacol. Ther..

[8] Rome Group for the Epidemiology and Prevention of Cholelithiasis (GREPCO) (1984). Prevalence of gallstone disease in an Italian adult female population. Am. J. Epidemiol..

[9] Barbara L., Sama C., Labate A.M., Taroni F., Rusticali A.G., Festi D., Sapio C., Roda E., Banterle C., Puci A. (1987). A population study on the prevalence of gallstone disease: The Sirmione Study. Hepatology.

[10] (1988). The Rome Group for Ep-idemiology and Prevention of Cholelithiasis (GREPCO). The epidemiology of gallstone disease in Rome, Italy. Part I. Prevalence data in men. Hepatology.

[11] Loria P.D., Dilengite M.A., Bozzoli M., Carubbi F., Messora R., Sassatelli R., Bertolotti M., Tampieri A., Tartoni P.L., Cassinadri M. (1994). Prevalence rates of gallstone disease in Italy. Eur. J. Epidemiol..

[12] Shaffer E.A. (2006). Epidemiology of gallbladder stone disease. Best Pr. Res. Clin. Gastroenterol..

[13] Angelico F., Del Ben M., Barbato A., Conti R., Urbinati G. (1997). Ten-year incidence and natural history of gallstone disease in a rural population of women in central Italy. The Rome Group for the Epidemiology and Prevention of Cholelithiasis (GREPCO). Ital. J. Gastroenterol. Hepatol..

[14] The Rome Group for Epidemiology and Prevention of Cholelithiasis (GREPCO) (1988). The epidemiology of gallstone disease in Rome, Italy. Part II. Factors associated with the disease. Hepatology.

[15] Biddinger S.B., Haas J.T., Yu B.B., Bezy O., Jing E., Zhang W., Unterman T.G., Carey M.C., Kahn C.R. (2008). Hepatic insulin resistance directly promotes formation of cholesterol gallstones. Nat. Med..

[16] Kim S.S., Lee J.G., Kim D.W., Kim B.H., Jeon Y.K., Kim M.R., Huh J.E., Mok J.Y., Kim S.-J., Kim Y.K. (2011). Insulin Resistance as a Risk Factor for gallbladder stone formation in Korean postmenopausal women. Korean J. Intern. Med..

[17] Nervi F., Miquel J.F., Alvarez M., Ferreccio C., García-Zattera M.J., González R., Pérez-Ayuso R.M., Rigotti A., Villarroel L. (2006). Gallbladder disease is associated with insulin resistance in a high risk Hispanic population. J. Hepatol..

[18] Di Ciaula A., Garruti G., Wang D.Q.-H., Portincasa P. (2018). Cholecystectomy and risk of metabolic syndrome. Eur. J. Intern. Med..

[19] Ata N., Kucukazman M., Yavuz B., Bulus H., Dal K., Ertugrul D.T., Yalcin A.A., Polat M., Varol N., Akin K.O. (2011). The metabolic syndrome is associated with complicated gallstone disease. Can. J. Gastroenterol..

[20] Lin I.-C., Yang Y.-W., Wu M.-F., Yeh Y.-H., Liou J.-C., Lin Y.-L., Chiang C.-H. (2014). The association of metabolic syndrome and its factors with gallstone disease. BMC Fam. Pract..

[21] Shen C., Wu X., Xu C., Yu C., Chen P., Li Y. (2014). Association of cholecystectomy with metabolic syndrome in a Chinese population. PLoS ONE.

[22] Chen L.-Y., Qiao Q.-H., Zhang S.-C., Chen Y.-H., Chao G.-Q., Fang L.-Z. (2012). Metabolic syndrome and gallstone disease. World J. Gastroenterol..

[23] Park Y., Kim D., Lee J.S., Na Kim Y., Jeong Y.K., Lee K.G., Choi D. (2017). Association between diet and gallstones of cholesterol and pigment among patients with cholecystectomy: A case-control study in Korea. J. Health Popul. Nutr..

[24] Van Erpecum K.J. (2011). Pathogenesis of cholesterol and pigment gallstones: An update. Clin. Res. Hepatol. Gastroenterol..

[25] Baratta F., Pastori D., Angelico F., Balla A., Paganini A.M., Cocomello N., Ferro D., Violi F., Sanyal A.J., Del Ben M. (2020). Nonalcoholic fatty liver disease and fibrosis associated with increased risk of cardiovascular events in a prospective study. Clin. Gastroenterol. Hepatol..

[26] Baratta F., Pastori D., Bartimoccia S., Cammisotto V., Cocomello N., Colantoni A., Nocella C., Carnevale R., Ferro D., Angelico F. (2020). Poor adherence to mediterranean diet and serum lipopolysaccharide are associated with oxidative stress in patients with non-alcoholic fatty liver disease. Nutrients.

[27] Grundy S.M., Cleeman J.I., Daniels S.R., Donato K.A., Eckel R.H., Franklin B.A., Gordon D.J., Krauss R.M., Savage P.J., Smith S.C. (2005). Diagnosis and management of the metabolic syndrome. Circulation.

[28] Zhu Q., Sun X., Ji X., Zhu L., Xu J., Wang C., Zhang C., Xue F., Liu Y. (2016). The association between gallstones and metabolic syndrome in urban Han Chinese: A longitudinal cohort study. Sci. Rep..

[29] Chen Y., Wu S., Tian Y. (2018). Cholecystectomy as a risk factor of metabolic syndrome: From epidemiologic clues to biochemical mechanisms. Lab. Investig..

