# Supplementary material for: Sex-Related Differences in the Association between Metabolic Syndrome and Gallstone Disease
Source: Int J Environ Res Public Health. 2021 Feb 18;18(4):1958. doi: 10.3390/ijerph18041958 (PMC7922698; doi:10.3390/ijerph18041958)
Supplement: Supplementary file 1 [file ijerph-18-01958-s001.pdf]

International Journal of
Environmental Research and Public Health

MDPI

Table S1. Univariate analysis of clinical and laboratory variables associated with gallstone disease and cholecystectomy in men.

Variables	Gallstone Disease		Cholecystectomy	
	**OR (95% CI)**	***p***	**OR (95% CI)**	***p***
**Age above median**	2.219 (1.209-4.072)	0.019	2.652 (1.057-6.659)	**0.038**
**Obesity (BMI≥30kg/m²)**	1.832 (1.017-3.300)	0.044	2.168 (0.915-5.135)	0.079
**Central Obesity (High waist circumference) ***	1.310 (0.727-2.361)	0.368	1.424 (0.613-3.310)	0.411
**Impaired Fasting Glucose ***	2.437 (1.327-4.475)	0.004	4.282 (1.577-11.776)	0.005
**Low HDL ***	1.302 (0.716-2.367)	0.386	2.579 (1.100-6.047)	0.029
**High triglycerides ***	1.072 (0.596-1.930)	0.817	0.707 (0.292-1.708)	0.441
**Total Cholesterol (mg/dl)**	0.994 (0.987-1.002)	0.163	0.993 (0.983-1.004)	0.193
**LDL Cholesterol (mg/dl)**	0.994 (0.986-1.005)	0.871	0.997 (0.985-1.009)	0.615
**High Blood Pressure ***	2.204 (0.952-4.300)	0.067	2.652 (0.772-9.109)	0.121
**Metabolic Syndrome**	2.248 (1.187-4.260)	0.013	5.743 (1.671-19.740)	0.006
**Metabolic Syndrome Score**	1.402 (1.110-1.772)	0.014	1.592 (1.117-2.269)	**0.010**
**High HOMA IR**	1.167 (0.632-2.154)	0.662	1.857 (0.761-4.531)	0.174
**High ALT ****	0.644 (0.344-1.204)	0.168	1.011 (0.427-2.398)	0.979
**High AST ****	0.831 (0.356-1.941)	0.669	1.173 (0.385-3.577)	0.122
**High GGT ****	1.090 (0.592-2.009)	0.728	1.716 (0.736-4.001)	0.211

* = defined according to ref. [26]; ** = values above median; CI = confidence interval; OR= odds ratio

Table S2. Univariate analysis of clinical and laboratory variables associated with gallstone disease and cholecystectomy in women.

Variables	Gallstone Disease		Cholecystectomy	
	**OR (95% CI)**	***p***	**OR (95% CI)**	***p***
**Age above median**	2.330 (1.144-4.349)	0.010	3.096 (1.344-7.131)	**0.008**
**Obesity (BMI≥30kg/m²)**	1.304 (0.702-2.419)	0.401	1.386 (0.680-2.823)	0.369
**Central Obesity (High waist circumference) ***	1.595 (0.522-4.873)	0.413	1.728 (0.915-3.264)	0.092
**Impaired Fasting Glucose***	0.887 (0.474-1.659)	0.707	0.825 (0.402-1.696)	0.602
**Low HDL***	0.818 (0.419-1.596)	0.556	0.951 (0.446-2.030)	0.898
**High triglycerides ***	0.992 (0.514-1.931)	0.992	1.004 (0.470-2.144)	0.992
**Total Cholesterol (mg/dl)**	1.006 (0.998-1.014)	0.120	1.002 (0.993-1.011)	0.719
**LDL Cholesterol (mg/dl)**	1.005 (0.997-1.014)	0.229	1.001 (0.991-1.011)	0.868
**High Blood Pressure ***	1.942 (0.933-4.041)	0.076	1.697 (0.732-3.933)	0.218
**Metabolic Syndrome**	1.265 (0.677-2.366)	0.461	1.519 (0.728-3.169)	0.265
**Metabolic Syndrome Score**	1.061 (0.836-1.346)	0.627	1.060 (0.806-1.394)	**0.678**
**High HOMA IR**	0.804 (0.406-1.592)	0.532	0.878 (0.406-1.898)	0.741
**High ALT ****	1.479 (0.755-2.897)	0.254	1.671 (0.789-3.541)	0.671
**High AST ****	1.401 (0.604-3.251)	0.423	2.348 (0.985-5.594)	0.054
**High GGT ****	1.763 (0.889-3.496)	0.104	2.323 (1.091-4.946)	0.029
